# Trajectories of postoperative serum troponin concentrations following pediatric heart transplantation

**DOI:** 10.1016/j.jhlto.2023.100039

**Published:** 2023-12-06

**Authors:** Alexander J. Kula, Erin Albers, Bora Hong, Mariska Kemna, Joshua Friedland-Little, Yuk Law

**Affiliations:** a Division of Nephrology, Ann & Robert H. Lurie Children’s Hospital of Chicago, and Department of Pediatrics, Northwestern University, Chicago, Illinois; b Department of Biostatistics, University of Washington, Seattle, Washington; c Division of Cardiology, Seattle Children's Hospital, and Department of Pediatrics, University of Washington, Seattle, Washington

**Keywords:** pediatric heart transplant, biomarkers, troponin, epidemiology, outcomes research

## Abstract

**Background:**

Troponin is a biomarker of myocardial injury and death but has not been well studied after pediatric heart transplants. The objective of this analysis is to describe the distribution and clinical determinants of serum troponin measured in the first week after pediatric heart transplantation.

**Methods:**

We included all patients who underwent heart transplantation at Seattle Children’s Hospital between 2012 and 2016. Serum Troponin-I (TnI) was measured daily in the first week after transplant. We described the distribution of serum TnI, and examined the relationship between peak TnI with known pre- peri-operative risk factors for myocardial injury including etiology of heart failure, ischemia time, and donor to recipient characteristics. Logistic regression models were used to test the association between peak TnI with incidence of death or rejection and formation of donor-specific antibodies (DSA) within 1 year. Adjusted models included age, HF etiology, crossmatch status, and panel reactive antibodies.

**Results:**

During the study period, 86 transplants were performed on 83 unique individuals. Serum TnI peaked at a median of 0.9 days after transplantation. In adjusted models, higher peak TnI was associated with death and/or rejection within 1-year post-transplant (odds ratio [95% confidence interval]: 1.10 [1.02, 1.19]). Peak TnI was not associated with de-novo DSA formation in adjusted models (OR [95%CI]: 1.01 [0.94, 1.09]). Post-transplant length of stay in the intensive care unit was positively correlated with peak TnI (*r* = 0.36, *p* < 0.001).

**Conclusions:**

This study describes serum TnI in the first week after pediatric heart transplant; a population for whom existing data are sparse. Our findings suggest TnI may have utility as a readily measurable biomarker of transplant-related myocardial injury. These results may inform future investigations of the prognostic significance of higher post-transplant TnI in future studies.

## Background

Concentrations of serum cardiac troponins (Tn) reflect myocardial damage secondary to cardiac strain, inflammation, or ischemia.^1^ Its utility as a diagnostic and prognostic biomarker has been well shown in both cardiac and noncardiac patient populations.^2^ The normally intracellular Tn only becomes found in the extracellular compartment with myocardial injury or death. This direct physiologic connection has also led to mechanistic insights to the processes driving cardiac disease. Despite the wide availability of Tn assays and its use as a diagnostic biomarker, one context to which there is not a clear description or applicability of Tn measurement is in pediatric heart transplantation.

For children with advanced heart failure (HF), receiving a heart transplant confers the best survival and long-term outcomes.^3^ Nonetheless, many factors compound the challenge of heart transplantation in children, including their small size, unique physiology, and limited number of transplant centers with pediatric expertise. Subsequently, the postoperative period requires intensive monitoring that includes radiographic, laboratory, and invasive testing. Identifying serum biomarkers can aid current cardiovascular monitoring and diagnostics, and potentially lead to better understanding of myocardial injury postheart transplant, allowing for earlier and more effective therapies.^4^, ^5^

Therefore, the objective of this analysis was to describe the trends and clinical determinants of serum troponin-I (TnI) in the first 7 days following heart transplantation in an exclusively pediatric population. We aimed to identify pre- and peri-operative factors which associate with higher postoperative TnI levels. Lastly, we performed a limited analysis of the prognostic significance of TnI within the first year following transplant.

## Methods

### Study population and data source

All patients who received a heart transplant between January 1, 2012, and December 31, 2016, at Seattle Children’s Hospital were included in this study. A total of 86 transplants on 83 patients were performed during this period. Only the first transplant was included for analysis for patients who received a retransplant within 12 months. Clinical data for those included in the study were extracted by direct import from the electronic health record (EHR) along with manual chart review. The study was approved by the Institutional Review Board at Seattle Children’s Hospital (STUDY00000567).

### Troponin-I collection and measurements

We analyzed serum TnI values collected as part of routine clinical care in the first 7 days following heart transplant. TnI levels were measured using the chemiluminescent microparticle immunoassay by Abbott Alinity.

Time-to-TnI measurements were classified both continuously and categorically. The continuous time-to-troponin measurement was calculated as the time between aortic cross clamp removal and troponin lab collection. Since the time of aortic cross-clamp removal was not always available in surgical records, it was estimated as occurring 10 minutes following the intraoperative transesophageal echocardiogram. A categorical time-to-troponin variable was created using the postoperative day (POD) of troponin collection, since this is frequently the conceptual framework often employed by those providing care. POD number 0 (POD #0) represented the time until 11:59 PM the same day as cross-clamp removal. POD #1 reflected the 24 hours following (midnight to midnight). The same process was repeated up to POD #7. Peak troponin represented the highest TnI value recorded within 7 days after heart transplant. For patients with only 1 measurement, this was classified as the peak value.

### Covariates

All baseline covariables were abstracted from the EHR at the time of transplant. This included demographic variables, etiology of heart failure (congenital heart defect vs cardiomyopathy), height, and weight. Donor-specific variables included donor age, height, weight, and cause of death. A binary panel reactive antibodies (PRA) variable was created and patients with a positive PRA of HLA-I or HLA-II antibody signal of any intensity were classified as positive. Similarly, patients were classified as having a positive crossmatch if there was reactivity to either T-cell or B-cell at any level of positivity. Ischemic time is the sum of cold plus warm time. Duration of cardiopulmonary bypass was collected from surgical records. Length of stay in the intensive care unit (ICU LOS) represents calendar days from arrival in the ICU postoperatively to discharge to floor or home. No participants died before discharge from ICU.

All patients received standard immunosuppression which consisted of high dose methylprednisolone in the first week followed by a taper over 6 weeks; mycophenolate mofetil started by POD 3; intravenous immunoglobulin 800 mg/kg given in the first week; antithymocyte globulin (Sanofi, USA) 5 mg/kg over 5 days in the first week; and tacrolimus typically initiated within the first week with a target of 10-12 ng/ml. The only exception to the use of antithymocyte globulin would be patients who went on ECMO or the chest was left opened which is rare as shown above and the use of antithymocyte globulin would be delayed, not fully withdrawn, until patient’s situation with extracorporeal membrane oxygenation (ECMO) or chest closure changed.

Post-transplant data were abstracted from the EHR and/or collected by manual chart review by the investigators. All patients had donor-specific antibody (DSA) monitoring, although the frequency and timing varied among patients. Patients were classified as having a positive DSA if they had mean fluorescence intensity >4000 at any locus. Rejection events were identified via chart review. We defined rejection in this study as events that were treated with rescue or significant augmentation of immunosuppression and when the biopsy showed ≥ ISHLT 1R for acute cellular rejection (ACR) or pathologic diagnosis of antibody mediatied rejection category 1 (pAMR1). In the cases where biopsy was not available or equivocal, the diagnosis was at the discretion of the treating heart failure attending given their synthesis of all available clinical information at that time.

### Statistical analysis

Patient and donor characteristics are reported as mean and standard deviation for normally distributed variables or as median and interquartile range for non-normally distributed data. Median peak and daily postop troponin values were calculated for all participants. Pearson correlation was used to compare clinical determinants with peak TnI. Mean peak TnI levels were compared between those with an ischemic time greater or less than 240 minutes, those with and without an etiology of congenital heart defect, and with a length of stay (LOS) in the ICU above or below the median using independent samples *t*-tests.

Logistic regression models were built to explore the association between peak serum troponin with a composite outcome of rejection or death within the first year. We focused outcome measures within 1-year following transplant as previous evidence suggests clinical trajectories in the first year following transplant are highly predictive of long-term outcomes.^6^, ^7^ Unadjusted and adjusted regression models testing the association between peak troponin and DSA formation were performed as secondary analyses. Adjusted models for all analyses included age, etiology of heart failure, crossmatch positivity, and PRA positivity. Odds-ratio along with 95% confidence intervals were reported for all models. For all analyses, a *p*-value <0.05 was considered statistically significant. All statistical analyses were conducted using SPSS Statistics version 26.0 (IBM Corp, Armonk, NY) or R, version 3.6.2 (R Core Team, 2019. R Foundation for Statistical Computing, Vienna, Austria).

## Results

### Baseline characteristics

A total of 448 troponin measurements were made over 86 transplants of 83 unique individuals. Baseline characteristics for this pediatric heart transplant cohort are presented in Table 1. The mean age was 6.6 years and 58% of the patients were male sex. Heart failure was secondary to a congenital heart defect etiology in 64% of patients. Donors were a mean 1.4× heavier than recipients and 1.1× taller. At transplant, only 6% of recipients had a positive crossmatch. The mean total ischemic time was 4.4 hours.

**Table 1:** Baseline Characteristics for Pediatric Heart Transplant Recipients Included in the Analysis

N = 83	
Factor	Value
*Recipient demographics*	
Age (years), mean ± SD	6.6 ± 6.6
Male sex, *n* (%)	48 (58%)
Height (cm), mean ± SD	104 ± 43
Weight (kg), mean ± SD	24 ± 22
Congenital etiology of heart failure, *n* (%)	53 (64%)
*Donor characteristics*	
Donor age (years), mean ± SD	9.2 ± 9.7
Donor Weight (kg), mean ± SD	30 ± 26
Donor Height (cm), mean ± SD	114 ± 42
Donor/recipient weight ratio, mean ± SD	1.4 ± 0.5
Donor/recipient height ratio, mean ± SD	1.1 ± 0.2
*Donor cause of death*	
Anoxia/hypoxia/other, *n* (%)	48 (58%)
Motor vehicle accident/head trauma (%)	35 (42%)
*Transplant surgery characteristics*	
Positive crossmatch *n* (%)	11 (6%)
Ischemic time (hour), mean ± SD	4.4 ± 1.6
Cardiopulmonary bypass time (hour), mean ± SD	2.6 ± 1.6

### Post-transplant Serum Troponin

**Figure 1:**
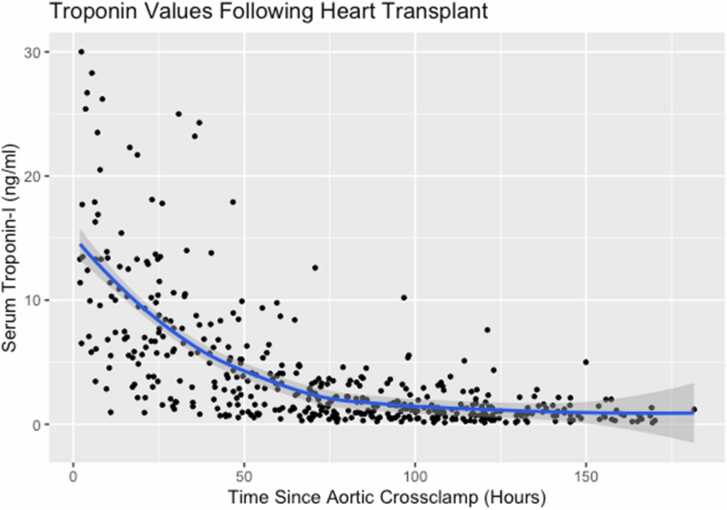
Trajectory of serum troponin values in the first 7 days after heart transplantation (*n* = 86).

Trends for postoperative TnI levels are presented in Tables 2 and Figure 1. Troponin levels peaked around 24 hours for most patients (median 0.9 days, IQR: 0.5, 1.1). Patients experienced a steep decline in TnI until around day 3, at which point the slope decreased. Rate of troponin decline had a close, inverse correlation with peak troponin value (*r* = −0.931, *p* < 0.001).

**Table 2:** Characteristics of Serum Troponin Levels in the First 7 Days After Heart Transplant (*n* = 86 Heart Transplants in *n* = 83 Individuals)

Variable	*n*	Median (IQR)
POD#0 serum troponin	15	13.5 (11.9, 22.6)
POD#1 serum troponin	75	7.5 (5.7, 12.8)
POD#2 serum troponin	74	4.9 (2.0, 7.4)
POD#3 serum troponin	79	2.3 (1.1, 3.7)
POD#4 serum troponin	79	1.5 (0.7, 2.1)
POD#5 serum troponin	78	1.0 (0.5, 1.6)
POD#6 serum troponin	40	0.9 (05, 1.4)
POD#7 serum troponin	23	0.6 (0.4, 0.8)
Peak serum troponin	86	9.4 (5.6, 13.5)
Peak time (days)	86	0.9 (0.5, 1.1)

### Pre/peri-operative associations with peak troponin

**Figure 2:**
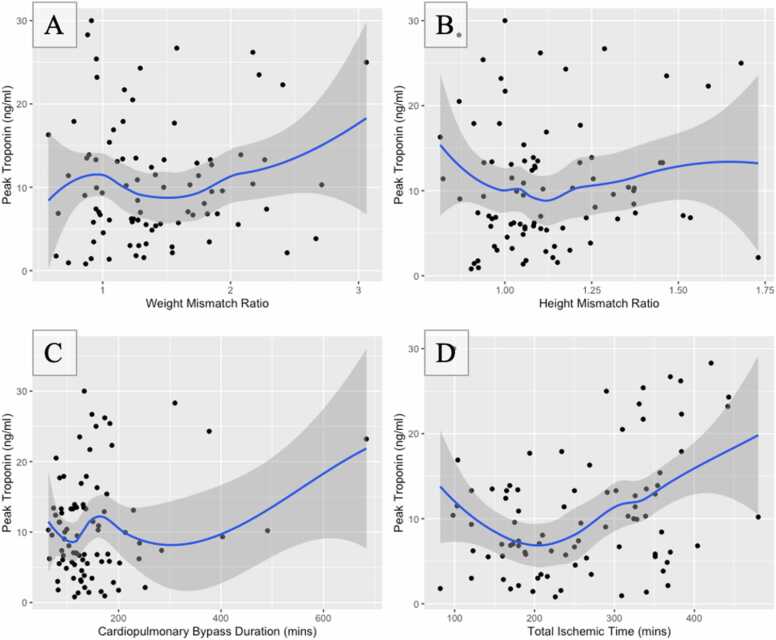
Relationship between donor-recipient weight (A) and height (B) ratios, cardiopulmonary bypass time (C), and total ischemic time (D) with peak serum troponin within the first 7 days after heart transplantation (*n* = 86).

**Figure 3:**
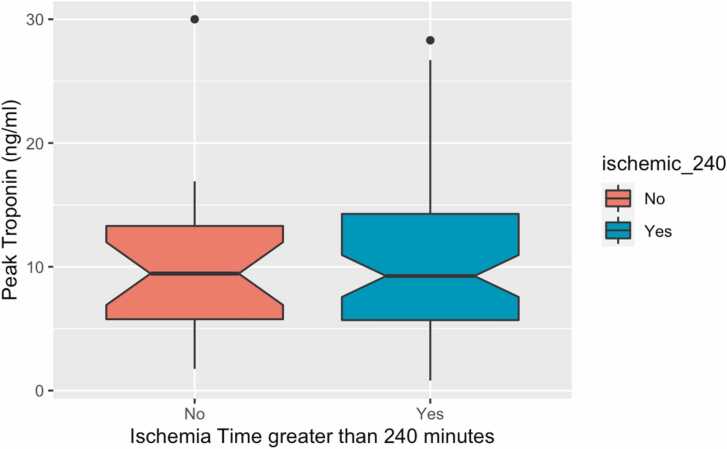
Mean peak serum troponin value in the first 7 days after heart transplantation for those with a total ischemic time greater or less than 240 minutes (*p* = 0.503).

**Figure 4:**
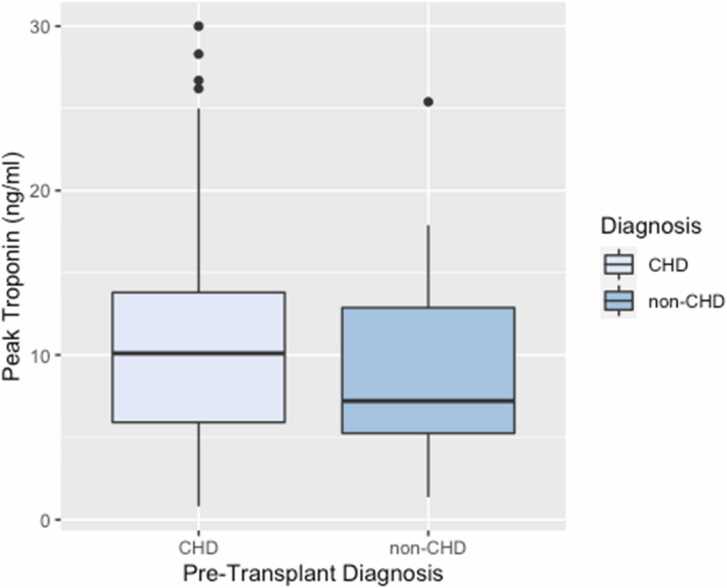
Mean peak serum troponin value in the first 7 days after heart transplantations stratified by heart failure etiology (*p* = 0.257).

We analyzed the relationship between pre- and peri-transplant variables and postoperative serum TnI levels (Figure 2, Figure 3, Figure 4). Figure 2 compares the extent of donor:recipient weight and height mismatch, cardiopulmonary bypass duration, and ischemic time with peak TnI values post-transplant. Longer ischemic times (R = 0.32, *p* = 0.003) and time on bypass (R = 0.22, *p* = 0.044) were correlated with peak TnI values, but a substantial amount of variation is present. Weight and height mismatch were not significantly correlated with peak TnI. Peak TnI levels were compared in Figure 3 for those with ischemic time dichotomized at 240 minutes (*p* = 0.503). Peak troponin values were not significantly different between these groups. Mean peak TnI values were higher in patients with a congenital heart defect, but this difference was not statistically significant (CHD etiology: 11.5 ng/ml, non-CHD etiology: 9.1 ng/ml; *p* = 0.117) Figure 4. There was a significant, positive correlation between peak troponin and ICU length of stay (*r* = 0.36, *p* < 0.001). The median postoperative LOS in the intensive care unit was 10 days. Patients with an ICU LOS > 10 days had a significantly higher peak troponin compared to those with <10 days ICU LOS (*p* < 0.001).

### Troponin and rejection and/or death

Rejection occurred in 23 (27%) of all transplants within the first year. The median [inter-quartile range] time to first rejection episode for all participants was 63 [20,146] days. No rejection event was recorded before day 6 postop. Details of rejection type and grade are included in Supplemental Table 1*.* There were 5 graft-losses, 4 of which were attributable to patient death. Of the 4 patients who died in the first year, the times to death were 18, 96, 114, and 116 days. Twenty-two patients developed positive DSA tests over the same timeframe. In unadjusted models, every doubling in peak serum troponin values was significantly associated with death and/or rejection within the first year (OR: 1.08; 95%CI: 1.01, 1.15) and had a positive, but nonsignificant association with de-novo DSA (OR: 1.01; 95%CI: 0.94, 1.08) (Table 3). In adjusted models, peak troponin was significantly associated with death and/or rejection (OR: 1.10; 95%CI: 1.02, 1.19).

**Table 3:** Association of Peak Serum Troponin Levels in the First 7 Days After Heart Transplant With Either Rejection or Death Within 1 Year by Logistic Regression

Odds ratio (95%CI) for death or rejection within <1 year after transplant		
	Unadjusted	Adjusted
Peak troponin (per doubling)	1.08 (1.01, 1.15)^a^	1.10 (1.02, 1.19)^a^

Odds ratio (95%CI) for DSA within <1 year after Transplant		

	Unadjusted	Adjusted

Peak troponin (per doubling)	1.01 (0.94, 1.08)	1.01 (0.94, 1.09)

Abbreviations: DSA, donor-specific antibodies.

Adjusted models include: age, diagnosis (congenital heart disease (CHD) or non-CHD), positive crossmatch, PRA.

a Denotes *p* < 0.05.

## Discussion

This observational study of serum troponin levels following pediatric heart transplant provides a novel description of its postoperative trends along with possible insights into the relationship between TnI and outcomes of clinical importance. Many peri-operative factors may contribute to elevated troponin levels, and serum troponin could serve as a more specific biomarker of myocardial stress related to transplantation, regardless of the etiology. Relatedly, patients with elevated post-transplant troponin levels may suggest elevated risk for rejection and other complications within 1 year that can lead to graft loss.

Contributions to immediate post-transplant troponin values are multifactorial. Serum troponin values can be elevated in heart donors prior to donation.^8^ Additionally, studies have shown that troponin concentrations from the perfusate during transport of the graft increase with time, and increased ischemia times in adults have been associated with increased peri-operative troponin values.^9^, ^10^ Our analysis also observed a significant correlation between ischemic time and peak troponin. Nonetheless, there was significant variability in peak troponin levels for any given ischemic time. This suggests that total ischemic time is just one of many factors contributing to peri-operative myocardial injury and subsequent troponin release. Other studies have noted that the conditions used for preservation also influence troponin release during transport.^9^, ^11^ We could not differentiate the relative contribution to peak troponin leak from procurement to the surgical implant phase, to early postoperative conditions in this single-center study. Nonetheless, the presence of myocardial injury represented by elevated troponin levels can serve as a target to improve the protection of the allograft at each of these phases perioperatively.

Our results are similar to findings in the adult literature noting the association between post-transplant troponin levels and adverse outcomes.^12^, ^13^ We noted a positive correlation between peak troponin and ICU length of stay. A similar finding was described in a study of post-transplant troponin in adult heart transplant patients.^14^ Other studies in adults suggest that elevated troponin levels in the first days following transplantation are associated with increased rates of early graft failure, acute kidney injury, and mortality.^9^, ^14^, ^15^, ^16^ Peak troponin values represent the summation of injury sustained in the peri-transplant period, although it is uncertain to what extent they represent ongoing myocardial injury in the postoperative period. Nearly all the patients in our study had their peak troponin values within 24 hours, followed by a steady decline. This observation can aid the design of other studies as future research in larger study populations, with precisely set time points for TnI collection, can better assess the utility of troponin and its ability to risk stratify and predict clinical outcomes.

The significance of elevated troponin levels following transplantation might also indicate those at increased risk beyond the initial hospitalization. We noted a modest association between elevated peak troponin and the risk for rejection and the development of DSAs within the first year. Most existing research has looked at the usefulness of serum troponin levels as noninvasive biomarkers to diagnose acute rejection.^17^, ^18^, ^19^ Results from these studies have been mixed and employed a wide variety of troponin assays and clinical contexts.^4^, ^20^ Our findings might be best interpreted using a different paradigm. Rather than elevated troponin being the result of myocardial injury secondary to rejection, we wonder whether elevated troponin levels associated with myocardial injury create an environment conducive to the development of future rejection. Myocardial injury sustained during transplant, as indicated by serum troponin, potentially could allow for immune recognition of otherwise privileged intracellular antigens. The amount of myocardial injury may also be commensurate with the extent of myocardial inflammation, such as from reperfusion injury. Along with allo-recognition and alloimmune response, these factors can converge to put the graft at risk for rejection later on. Perhaps, the observation of an association with positive DSAs (though not statistically significant in this sample size) is a further suggestion of this augmented alloimmune response. Given the multitude of clinical and immunologic factors present surrounding heart transplantation, more research is required to explore the significance of peri-transplant myocardial injury and allograft-specific immunologic activation.

Strengths of this study include the availability of detailed clinical data relating to heart transplantation and 1-year follow-up. TnI measurement occurred using clinical laboratory equipment with frequent quality control assessments. While the volume of heart transplantation at any one center is limited, we were able to create a slightly larger study population by utilizing data from all heart transplants over a 5-year period. The collection of TnI was part of a clinical protocol for all transplants. We also acknowledge the presence of weaknesses in this analysis. The TnI collection was not part of a research protocol where monitoring was performed to ensure its collection or at precise time points from the release of cross-clamp, but at each morning postoperatively. We did not have data beyond 1-year post-transplant nor TnI in donors pretransplantation. Due to limitations in the data, we could only identify total ischemia time and could not reliably quantify separately the cold vs warm ischemia time. This should be followed up in future studies, as it could be a determinant of the variability between peak TnI and total ischemic time*.* We were not able to reliably identify patients with primary graft dysfunction (PGD) through chart review. Future studies must examine the association between postoperative troponin and risk for PGD, and whether PGD is a potential mediator of the association between elevated peak troponin and risk for rejection. The size of our study population could have reduced our power to identify statistically significant predictors and related outcomes of peak troponin levels. We attempted to minimize confounding by using potentially relevant covariables; however, given the complexity surrounding heart transplant, the risk for confounding cannot be eliminated. Relatedly, this is an observational study, so causality cannot be determined. Since only one transplant center was included, the generalizability of some of our findings may have limitations.

This observational study provides a description of the trends and associations of TnI levels in the first week following heart transplant. Post-transplant TnI most often peaks in the first day after transplantation, decreases quickly until POD#3, at which point the rate of decline decelerates. We noted a possible association between higher peak troponin and rejection within the first year, but this association needs to be further elucidated. These observations can aid the design of future research, using larger study populations, to more formally assess the utility of postoperative troponin to risk-stratify and predict adverse outcomes.

## Disclosure statement

The authors declare that they have no known competing financial interests or personal relationships that could have appeared to influence the work reported in this paper.
