# Supplementary material for: Trajectories of postoperative serum troponin concentrations following pediatric heart transplantation
Source: JHLT Open. 2023 Dec 6;5:100039. doi: 10.1016/j.jhlto.2023.100039 (PMC11935518; doi:10.1016/j.jhlto.2023.100039)
Supplement: Supplementary file 1 — Supplementary material [file mmc1.docx]

Trajectories of Post-Operative Serum Troponin Concentrations Following Pediatric Heart Transplantation

Alexander J Kula, Erin Albers, Bora Hong, Mariska Kemna, Joshua Friedland-Little, Yuk Law.

Supplemental Appendix

**Supplemental Table 1: Characteristics of all rejection episodes that occurred within 1 year of transplant.**

|  | Acute Cellular Rejection | Antibody Mediated Rejection |
| --- | --- | --- |
| Total Number (% of rejection) | 13 (59%) | 9 (41%) |
| Grade 1, n (% of ACR/AMR) | 0 (0%) | 6 (67%) |
| Grade 2, n (%) | 10 (77%) | 2 (22%) |
| Grade 3, n (%) | 2 (15%) | 0 (0%) |
| Clinically without biopsy , N (%) | 1 (8%) | 1 (11%) |

ACR: Acute Cellular Rejection, AMR: Antibody Mediated Rejection
